# Spray-induced gene silencing for plant disease control: mechanistic basis and deployment-oriented design

**DOI:** 10.3389/fpls.2026.1829123

**Published:** 2026-05-18

**Authors:** Mohamed Mannaa, Eui-Joon Kil, Inmyoung Park, Young-Su Seo

**Affiliations:** 1 Department of Microbiology, Pusan National University, Busan, Republic of Korea; 2 Department of Plant Pathology, Cairo University, Faculty of Agriculture, Giza, Egypt; 3 Department of Plant Medicals, Gyeongkuk National University, Andong, Republic of Korea; 4 School of Food and Culinary Arts, Youngsan University, Busan, Republic of Korea; 5 Institute of Systems Biology, Pusan National University, Busan, Republic of Korea

**Keywords:** crop protection, cross-kingdom RNAi, double-stranded RNA, nanocarriers, RNA interference, spray-induced gene silencing

## Abstract

Spray-induced gene silencing (SIGS) is a transgene-free, biodegradable RNA-based approach for crop protection in which exogenously applied double-stranded RNA (dsRNA) is taken up and processed by plants or susceptible pathogens to drive the sequence-specific silencing of essential or virulence-associated genes. This review synthesizes advancements in transitioning SIGS from proof-of-concept to field-relevant deployment. Here, we frame SIGS as a two-compartment process: (i) dsRNA deposition on foliage, access to cuticular/apoplastic microenvironments, processing into small interfering RNAs (siRNAs), amplification, and systemic movement within plants; and (ii) pathogen acquisition of dsRNA/siRNAs at infection interfaces, followed by RNA interference (RNAi) execution, with outcomes strongly conditioned by pathogen RNA-uptake competence and, in some systems, cross-kingdom RNA trafficking. Because performance is often constrained by exposure rather than sequence potency, we evaluated the key determinants of delivery and persistence and compared carrier strategies that extend stability and bioavailability, including layered double hydroxide clays, vesicle-inspired lipid systems, polymer complexes, and carbon-based nanomaterials. We then consolidated the mechanism-informed design rules for target selection, within-transcript positioning, and dsRNA architecture, along with specificity, non-target risk, and durability/escape management. Finally, we defined the current scope boundaries, including the limited applicability to bacterial phytopathogens lacking canonical eukaryotic RNAi. We outline deployment-oriented priorities for achieving reliable SIGS performance under realistic agricultural conditions.

## 1 Introduction

Plant diseases remain among the most serious constraints on global food security, as they reduce crop yield, quality, and resilience across nearly all agricultural systems. Their increasing impact reflects converging pressures, including intensified monoculture, global trade in plant materials, and climate-driven shifts in pathogen distribution and virulence, which elevate epidemic frequency and severity with substantial agronomic and economic consequences (Fones et al., 2020; Strange and Scott, 2005). These trends challenge food production stability and intensify the need for effective and environmentally compatible disease management strategies (Savary et al., 2019).

Chemical pesticides, including fungicides with distinct modes of action such as multi-site inhibitors and site-specific compounds, have historically dominated crop protection due to their rapid and broad-spectrum activities. However, prolonged and intensive pesticide use has resulted in high ecological and evolutionary costs, including the selection of resistant pathogen populations, disruption of beneficial organisms and ecosystem functions, and contamination of soil and aquatic environments (Aktar et al., 2009). In fungicides, mode-of-action differences are also relevant to resistance risk, with site-specific fungicides generally being more vulnerable to resistance development than multi-site compounds. In addition, fungicide residues can affect non-target organisms and freshwater ecosystems, reinforcing the need for next-generation plant protection approaches that reduce chemical inputs without compromising efficacy.

Among emerging alternatives, RNA-based crop protection—particularly spray-induced gene silencing (SIGS) achieved through topical application of double-stranded RNA (dsRNA)—has gained attention as a precise, biodegradable, and non-transgenic strategy for disease suppression (Dubrovina and Kiselev, 2019; Liu et al., 2024b). SIGS exploits RNA interference (RNAi) to achieve the sequence-specific silencing of target transcripts following dsRNA uptake and processing in plants and/or interacting pathogens. Unlike host-induced gene silencing (HIGS), which depends on transgenic expression of RNA constructs within the plant and endogenous production of silencing molecules during host–pathogen interaction, SIGS relies on the exogenous application of dsRNA to plant surfaces. This topical mode of delivery makes SIGS a non-transgenic and more rapidly retargetable strategy for emerging or evolving pathogens while retaining RNA-guided specificity (Cagliari et al., 2019).

These two biological processes underlie the feasibility of SIGS in multiple pathosystems. First, cross-kingdom RNA trafficking between plants and associated organisms, often via extracellular vesicle–mediated routes, provides a mechanistic basis for RNA movement across species boundaries (Cai et al., 2018). Second, many eukaryotic pathogens exhibit environmental RNA uptake, enabling internalization of exogenous dsRNA and downstream gene silencing, as demonstrated in diverse fungi, including *Sclerotinia sclerotiorum* (Wytinck et al., 2020; Šečić and Kogel, 2021). Together, these features make SIGS particularly relevant to fungal and oomycete pathogens, where pathogen-side uptake and RNAi execution can often be interrogated directly, and to antiviral protection, where plant RNA silencing provides the principal effector framework (Cai et al., 2018; Qi et al., 2024).

These requirements for cross-kingdom RNA trafficking and environmental RNA uptake by susceptible eukaryotic pathogens also define the current limitations of SIGS applicability. Most bacterial phytopathogens lack canonical eukaryotic RNAi machinery, including Dicer-mediated small interfering RNAs (siRNA) processing and Argonaute–RNA-induced silencing complex (RISC)–guided transcript knockdown, thereby restricting the potential for true RNAi-based suppression by exogenous dsRNA (Shabalina and Koonin, 2008; Willkomm et al., 2015). Although prokaryotic Argonaute systems are present in some bacteria, they primarily function in nucleic acid defense and do not replicate the RNAi pathways exploited by SIGS (Swarts et al., 2014; Willkomm et al., 2018). Consequently, current SIGS applications are most directly applicable to fungal and oomycete diseases and plant viral infections, whereas bacterial disease control requires alternative molecular strategies.

As SIGS moves from proof-of-concept to field-relevant use, performance is increasingly determined by whether bioactive RNA exposure is achieved at the correct infection interfaces and for a sufficient duration, rather than by sequence potency alone (Rank and Koch, 2021). This exposure constraint is tightly coupled to the pathogen’s RNA uptake competence and how dsRNA is designed and formulated. Specifically, it depends on the selected target class, the region of the dsRNA transcripts, and how dsRNA architecture and delivery are engineered to maintain bioavailability under realistic conditions (Chen et al., 2025).

In this review, we synthesize recent advances in exogenous dsRNA-based SIGS as a next-generation, transgene-free strategy for plant disease control through a mechanism- and deployment-centered lens. Rather than surveying all pathogen classes with equal descriptive depth, we organize the review around the major determinants that govern SIGS performance across pathosystems: (i) plant-side deposition, entry, processing, and movement of sprayed RNA; (ii) pathogen-side acquisition, uptake competence, and RNAi execution at infection interfaces; and (iii) formulation, target selection, construct design, specificity, and durability as translational constraints on field-relevant efficacy. Within this framework, fungal and oomycete systems are discussed in greater mechanistic detail where pathogen uptake and RNAi execution are currently most directly resolved, whereas antiviral examples are used to illustrate plant-side RNA processing, systemic spread, and prophylactic deployment logic. This structure is intended to provide a clearer and more coherent basis for interpreting where SIGS is mechanistically supported, where its current scope boundaries lie, and which factors most strongly shape reliable deployment under realistic agricultural conditions. This review is designed to integrate mechanism and deployment within a single framework centered on exposure, uptake competence, target design, specificity, and durability as co-determinants of field-relevant efficacy.

## 2 Mechanistic basis of spray-induced gene silencing in plant–pathogen systems

**Figure 1:**
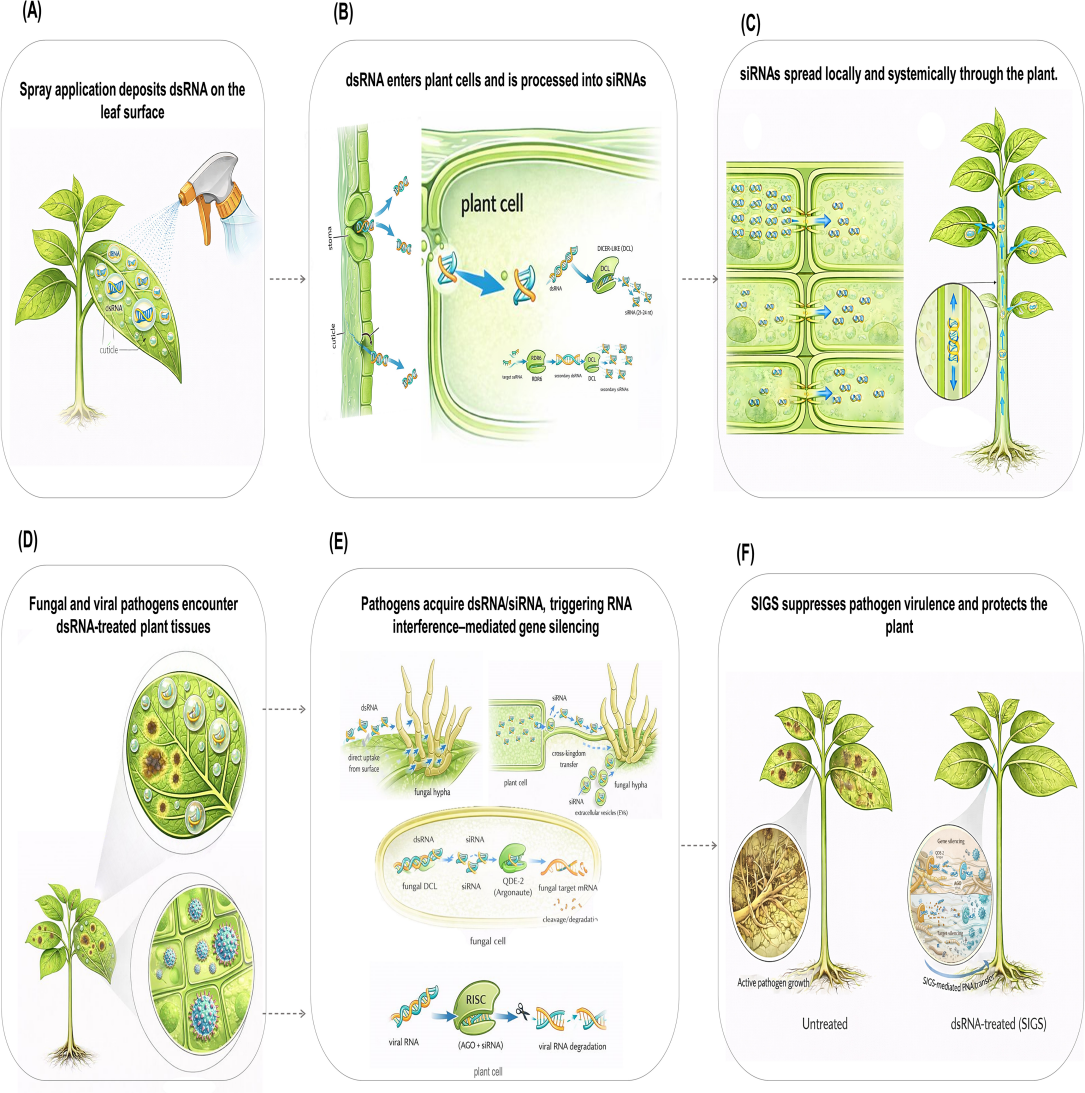
Conceptual overview of spray-induced gene silencing (SIGS) in plant protection. Exogenously applied dsRNA is deposited on the leaf surface **(A)**, gains access to plant tissues and is processed into siRNAs in plant cells **(B)**, and may spread locally or systemically within the plant **(C)**. At infection interfaces, pathogens encounter sprayed dsRNA and/or plant-derived small RNAs **(D)**, which may be acquired by uptake-competent fungal pathogens or contribute to plant-side antiviral RNAi responses **(E)**. Collectively, these processes can reduce pathogen gene expression and disease development, resulting in improved plant protection relative to untreated plants **(F)**.

Spray-induced gene silencing engages endogenous RNA-silencing pathways in plants and/or target pathogens after exogenous dsRNA is applied to plant surfaces (Koch et al., 2016; Kaldis et al., 2018; Basso et al., 2025). Mechanistically, SIGS is best viewed as a two-compartment process (**Figure 1**): (i) plant-side deposition, access, intracellular processing, and movement of sprayed RNA within host tissues, and (ii) pathogen-side acquisition of dsRNA and/or plant-derived small RNAs at infection interfaces, followed by RNAi execution where the pathogen is uptake-competent and encodes the relevant silencing machinery (Koch et al., 2016; Kaldis et al., 2018; Werner et al., 2020; Cai et al., 2018). The relative contribution of these compartments varies across pathosystems. In antiviral applications, the major effector phase is typically plant-side RNAi directed against viral RNA in planta, whereas in fungal and oomycete systems, performance often depends more directly on whether the pathogen can acquire environmental or host-derived RNA and execute gene silencing internally (Kaldis et al., 2018; Koch et al., 2016; Werner et al., 2020; Qiao et al., 2021). This distinction provides a useful framework for interpreting both mechanistic evidence and translational performance across SIGS systems.

### 2.1 Plant-side entry, intracellular processing, and spread of sprayed RNA

Following foliar application, dsRNA is first deposited onto aerial plant surfaces, where its immediate fate is determined by cuticular properties, hydration status, and microscale surface heterogeneity. Rather than entering uniformly, sprayed RNA partitions into cuticular and epidermal microenvironments and may gain access through natural openings or discontinuities, including stomata, hydathodes, trichome bases, wounds, and hydrated surface defects, or by diffusion into apoplastic spaces when local hydration conditions permit solubilization and movement (Dalakouras et al., 2020; Eichert and Goldbach, 2008; Brosnan et al., 2026). Thus, the first mechanistic barrier in SIGS is not sequence recognition, but physical access of sprayed dsRNA to permissive entry microenvironments.

After gaining access through natural surface openings or diffusion into apoplastic spaces, dsRNA can reach internal tissues, where it is internalized and processed by plant RNA-silencing machinery. In this context, DICER-LIKE (DCL) proteins cleave dsRNA into siRNAs, which are then loaded into ARGONAUTE (AGO)-containing RISC to guide sequence-specific cleavage or translational repression of complementary RNAs. Plant antiviral silencing provides the clearest mechanistic example of this process: DCL4 and DCL2 generate 21-nt and 22-nt siRNAs, respectively, and AGO2 is a major functional effector in restricting viral accumulation (Deleris et al., 2006; Harvey et al., 2011). In antiviral SIGS, this plant-side processing route provides the primary biochemical basis by which sprayed dsRNA is converted into an active siRNA pool capable of suppressing viral RNA in planta (Holeva et al., 2021; Vadlamudi et al., 2020).

Plant RNA silencing can also extend beyond the initial site of deposition and processing. RNA-dependent RNA polymerases, particularly RDR6 in many systems, can amplify silencing output by generating secondary dsRNA and secondary siRNAs from targeted transcripts, while mobile RNA species can spread locally through plasmodesmata and systemically via vascular tissues (Chen et al., 2010; Molnar et al., 2010). In this context, it is more precise to refer to the spread of sprayed dsRNA and/or derived siRNA pools, rather than to an undefined “silencing signal.” Evidence from barley shows that exogenously applied RNA can move beyond treated regions, indicating that SIGS may generate spatially extended RNA exposure profiles; however, systemic detection does not necessarily imply uniform bioactivity across tissues, because biological relevance still depends on tissue accessibility, RNA persistence, and whether pathogens encounter and internalize RNA at the relevant infection site (Biedenkopf et al., 2020).

### 2.2 Pathogen acquisition, RNAi execution, and cross-kingdom transfer at infection interfaces

The pathogen-side phase of SIGS is most directly resolved in fungal and oomycete pathosystems, where disease suppression often depends on whether the invading organism can acquire sprayed dsRNA and/or plant-derived small RNAs at the infection interface and then execute RNAi internally. Two nonexclusive routes are typically considered. In environmental RNAi, the pathogen directly internalizes extracellular dsRNA or siRNAs from the plant surface, apoplast, or other exposed microenvironments and processes them through its own Dicer/Argonaute machinery. In plant-mediated routing, sprayed dsRNA is first processed in planta, and the pathogen is subsequently exposed to host-derived small RNAs or mixed RNA pools at the host–pathogen boundary. The balance between these routes likely varies with host species, tissue context, infection stage, and formulation type (Werner et al., 2020; Cai et al., 2018; Schlemmer et al., 2022).

A defining mechanistic determinant is pathogen RNA-uptake competence, which is not uniform across taxa, isolates, or developmental stages. In a comparative analysis across multiple fungi and an oomycete, efficient dsRNA uptake was observed in pathogens such as *Botrytis cinerea*, *Sclerotinia sclerotiorum*, *Rhizoctonia solani*, *Aspergillus niger*, and *Verticillium dahliae*, whereas uptake was weak or not detected in others, and SIGS efficacy tracked these differences in uptake behavior (Qiao et al., 2021). In Fusarium head blight systems, spray-mediated silencing also depended on functional pathogen-side silencing machinery; dsRNAs targeting *FgAGO* and *FgDCL* genes reduced disease and supported genuine fungal RNAi execution rather than an exclusively plant-driven effect (Werner et al., 2020). Accordingly, antifungal SIGS performance is best understood as a function of two coupled requirements: sufficient RNA exposure at the infection interface and adequate pathogen capacity to internalize and process the encountered RNA into effective silencing outcomes. In addition to direct environmental uptake, cross-kingdom RNA trafficking may contribute to SIGS outcomes by moving regulatory RNA species across the host–pathogen boundary. A landmark study showed that plants can deliver small RNAs to fungal pathogens through extracellular vesicles (EVs), providing direct evidence that plant-derived RNA cargo can suppress fungal virulence genes during infection (Cai et al., 2018). Subsequent work further showed that RNA-binding proteins contribute to selective small-RNA loading into plant EVs and that EV-associated cargo may extend beyond sRNAs to other RNA species, including plant mRNAs with potential regulatory effects in fungal recipients (He et al., 2021; Wang et al., 2024). At the same time, EV-mediated routing should not be treated as the sole or uniformly dominant explanation for SIGS across pathosystems. For example, Schlemmer et al. (2022) showed that extracellular vesicles isolated from dsRNA-sprayed barley contained spray-derived siRNAs, but when these EV preparations were applied to *Fusarium graminearum* under the tested conditions, they did not induce detectable growth inhibition or target-gene silencing. These results suggest that EV-mediated transfer may contribute to, rather than universally define, the effective RNA pool at infection interfaces. This more limited role of EV-mediated transfer is consistent with the broader view that SIGS performance depends strongly on the composition of the effective RNA pool available at the infection interface and on pathogen-specific uptake behavior (Qiao et al., 2021).

Taken together, the available evidence supports interpreting SIGS as a convergent mechanistic network rather than a single linear route. Sprayed dsRNA may remain localized at surface or apoplastic infection interfaces, enter plant tissues and be processed into siRNAs, spread locally or systemically as dsRNA and/or derived small-RNA pools, and in some systems cross the host–pathogen boundary through EV-associated or related trafficking routes. Which of these processes most strongly determines efficacy depends on the pathosystem. In antiviral applications, plant-side processing and effector activity are often dominant, whereas in fungal and oomycete systems, realized performance more often hinges on interface exposure and pathogen uptake competence. From a translational perspective, the most informative mechanistic checkpoints are therefore access, processing, and uptake competence, because together they determine whether sufficient bioactive RNA reaches the relevant biological site for disease attenuation.

## 3 Delivery, formulation, and environmental persistence of sprayed dsRNA

A central translational challenge for SIGS is that exogenously applied dsRNA must remain intact and bioavailable long enough to access permissive plant microenvironments and/or persist at infection interfaces where pathogens can encounter and internalize RNA (Rank and Koch, 2021; Mitter et al., 2017). In practice, performance is constrained less by sequence potency than by realized exposure: how much RNA is delivered to, retained within, and maintained in a bioactive form at the relevant biological “contact points” (Imran et al., 2025). Loss pathways include rapid physicochemical partitioning on leaf surfaces, limited penetration across the cuticle–cell wall continuum, and environmental dissipation driven by solar radiation, rainfall/runoff, and nuclease- and microbe-mediated degradation (Parker et al., 2019). Therefore, the formulation functions as an exposure-control layer that shapes both efficacy and environmental fate, as discussed later.

### 3.1 Exposure constraints on leaf surfaces and at infection interfaces

After foliar spraying, dsRNA is not expected to distribute uniformly across the leaf surface; rather, it partitions into heterogeneous surface microhabitats shaped by wax chemistry, surface roughness, trichomes, stomatal complexes, and microcracks. This heterogeneity creates strong spatial variations in droplet retention, drying kinetics, and rehydration dynamics, which in turn govern the availability of dsRNA for entry routes or its loss through runoff and degradation (Fernández and Eichert, 2009; Hoang et al., 2022). Consequently, the biologically effective dose can decline rapidly even when nominal application rates appear adequate, particularly if RNA is not retained long enough to reach permissive microenvironments (e.g., hydrated discontinuities and stomatal-associated regions) or temporally overlaps with early infection events (Hoang et al., 2022).

Topical RNAi studies consistently reflect this exposure logic: unformulated (“naked”) dsRNA can be effective in receptive systems, but field-relevant protection often decays within a short window, motivating protective carriers and rainfast formulations designed to stabilize RNA and extend functional lifetime on leaves (Mitter et al., 2017; Niño-Sánchez et al., 2022; Spada et al., 2025). In other words, the formulation should be treated as a design variable coequal to target selection because it largely determines whether the sprayed dsRNA reaches and remains at biologically meaningful contact points. Thus, even when dsRNA sequences are intrinsically potent, the effective dose available for plant entry or pathogen encounter can fall rapidly after spraying, making retention, wetting behavior, rehydration, and persistence central determinants of field-relevant efficacy.

### 3.2 Environmental persistence, dissipation, and implications for efficacy and biosafety

Environmental fate is a defining constraint for SIGS, because sprayed dsRNA is inevitably exposed to dissipation processes on leaf surfaces and, via wash-off and plant residues, can enter soil and aquatic compartments (Dubelman et al., 2014). In the soil, dsRNA readily adsorbs to mineral and organic components, and its degradation can proceed via both abiotic and biotic pathways, rapidly reducing the pool of intact RNA (Dubelman et al., 2014; Bachman et al., 2020). Importantly, adsorption does not simply eliminate dsRNA; it may also alter its mobility and bioavailability, which could in turn shape exposure profiles relevant to both efficacy (where RNA availability for uptake is important) and biosafety (where non-target exposure is considered).

The formulation chemistry can measurably shift these profiles. In particular, cationic nanocarriers, including polymer-based systems, can enhance dsRNA persistence by electrostatically complexing with RNA, thereby limiting nuclease accessibility and reducing susceptibility to environmental degradation (Yang et al., 2022). Across compartments (soil, water, and plant tissues), dissipation datasets indicate that the applied dsRNA is generally transient, but persistence is context-dependent and can be extended by protective formulations. For example, LDH-based BioClay prolonged antiviral protection beyond the brief post-spray window observed for naked dsRNA, and BioClay formulations also extended protection against *Botrytis cinerea* under foliar application conditions (Mitter et al., 2017; Niño-Sánchez et al., 2022). The same property is exploited to achieve agronomically meaningful protection windows (Spada et al., 2025). Accordingly, persistence should not be viewed only as a technical advantage; rather, it can be interpreted as a coupled efficacy-and-exposure trait, because the same formulation properties that prolong functional RNA availability may also extend environmental residence and non-target exposure windows.

### 3.3 Formulation strategies to improve realized exposure, stability, and functional longevity

Formulation strategies for SIGS are most useful when viewed as solutions to specific exposure bottlenecks rather than as independent material classes. Some platforms mainly protect dsRNA from degradation and extend residence time, whereas others also improve interfacial presentation, internalization, or controlled release. Across systems, the most informative comparisons are therefore not material-versus-material in the abstract, but formulated versus naked dsRNA under biologically relevant conditions. For this reason, formulation is treated here as the broader translational concept, whereas individual carrier platforms are discussed as representative implementations of that concept.

#### 3.3.1 Mineral nanocarriers for RNA delivery: layered double hydroxide “BioClay”

Layered double hydroxide (LDH) nanosheets (BioClay) represent one of the most extensively characterized mineral carriers for extending the functional persistence of foliar-applied dsRNA/siRNA. LDH binds RNA through electrostatic complexation and intercalation within positively charged mineral layers, physically shielding RNA and slowing loss, while maintaining a pool of bioactive molecules available for uptake and processing at plant–pathogen interfaces (Ladewig et al., 2009). The foundational BioClay study established a “depot-and-release” principle: LDH-loaded dsRNA provided substantially longer antiviral protection than naked dsRNA following a single application, attributed to gradual, moisture-dependent release that sustained RNA availability beyond the brief post-spray window (Mitter et al., 2017).

Subsequent studies have indicated that LDH-enabled persistence is not limited to antiviral contexts. BioClay-based formulations have prolonged RNAi-mediated protection against *B. cinerea* (Niño-Sánchez et al., 2022) and have been used as topical delivery platforms in Fusarium crown and root rot of tomato to interrogate pathogenicity gene function under spray-based delivery conditions (Mosa and Youssef, 2021). These studies support the view that LDH platforms can convert a short-lived surface pulse into a longer exposure profile that better overlaps with the infection timing, thereby increasing the probability that sufficient RNA is available when and where silencing must occur.

Beyond protection, LDH may also increase effective delivery by extending RNA residence near discontinuous entry routes. Foliar uptake of hydrophilic macromolecules is constrained by surface heterogeneity and by temporally restricted uptake “windows” associated with hydration-dependent pathways (Eichert and Goldbach, 2008). In this setting, LDH can act as a protective reservoir, maintaining localized RNA availability until entry becomes feasible. Experimental studies have shown that clay nanoparticle systems can deliver small RNAs into intact leaf cells and induce functional knockdown without invasive procedures (Yong et al., 2022). From a product development perspective, LDH performance can be tuned via compositional and physicochemical variables (charge characteristics, binding strength, loading capacity, and release behavior) that directly affect foliage persistence and bioactivity (Ladewig et al., 2009). Because the realized dose also depends on wetting, spreading, adhesion, and rainfastness, LDH platforms are best optimized as integrated formulations in which carrier properties and spray adjuvants are co-developed to harmonize deposition, on-leaf stability, release kinetics, and uptake opportunities (Forster and Kimberley, 2015; Ladewig et al., 2009). This practical importance is reflected in studies where BioClay formulations prolonged antiviral protection after a single application and extended RNAi-mediated protection against *Botrytis cinerea* under foliar conditions, consistent with the view that on-leaf retention and controlled release are major contributors to realized efficacy (Mitter et al., 2017; Niño-Sánchez et al., 2022).

#### 3.3.2 Vesicle-inspired and lipid-based carriers

Vesicle-inspired lipid carriers (liposomes, lipid nanoparticles, and engineered nanovesicles) have been developed to address two coupled constraints: rapid extracellular loss of dsRNA on the phylloplane and inefficient access to compartments where plant processing and/or pathogen uptake can occur (Chen et al., 2023). By packaging dsRNA within membrane-like assemblies, these systems can shield RNA from destabilizing factors while improving interfacial compatibility with biological membranes, thereby increasing the likelihood that dsRNA remains available through drying–rehydration cycles and during hydration-dependent entry windows (Hoang et al., 2022). For fungal SIGS, this approach aligns with evidence that endocytosis contributes to environmental RNA uptake in multiple phytopathogenic fungi, implying that membrane-compatible formulations can be mechanistically relevant rather than merely preservative (Šečić and Kogel, 2021).

A clear demonstration is that the artificial nanovesicle platform applied against *B. cinerea*, a vesicle-loaded dsRNA delivered by foliar spray, produced a substantially longer protection window than naked dsRNA in tomatoes and grapes, with efficacy reported for up to several weeks (Qiao et al., 2023). Beyond prolonging integrity, lipid carriers can also influence the presentation of dsRNA at the plant–pathogen boundary by concentrating RNA in hydrated microdomains, reducing unproductive adsorption to leaf matrices, and supporting membrane-associated delivery steps that are otherwise inefficient for large polyanionic RNAs (Hoang et al., 2022). Conceptually, this strategy parallels the biological observation that plants deploy extracellular vesicles carrying regulatory RNAs during interactions with fungal pathogens (Cai et al., 2018). However, engineered lipidic carriers are distinct from native EVs. Key translational questions now center on performance robustness across variable microclimates and canopy architectures, manufacturability, spray stability, and cost-effectiveness at scale.

#### 3.3.3 Carbon-based nanomaterials: carbon dots and related platforms

Carbon-based nanomaterials, particularly carbon dots (CDs), have emerged as versatile SIGS carriers because their size, surface charge, and hydrophilicity can be tuned to influence the leaf-surface behavior, cellular entry, and local/systemic movement of nucleic acids (Schwartz et al., 2020; Wang et al., 2025; Venu et al., 2025). Schwartz et al. (2020) showed that very small CDs could deliver siRNAs across the plant cell wall in *Nicotiana benthamiana* and tomatoes, supporting the concept that engineered carbon nanostructures can function as transfection-like agents rather than as passive surface stabilizers.

Delgado-Martín et al. (2022) reported that CD–dsRNA nanocomposites sprayed onto cucumbers markedly increased internal dsRNA accumulation (~50-fold relative to naked dsRNA) and elevated siRNAs derived from the sprayed dsRNA (~13-fold), with enhanced levels detected systemically in the distal leaves. Hybrid strategies further extend this concept; formulations combining CDs with polyethylenimine-functionalized mesoporous silica nanoparticles increased *in planta* dsRNA accumulation and improved spray-induced silencing of both RNA and DNA viruses (Zarrabi et al., 2026). Carbon quantum dots have also been applied to oomycetes and fungal targets; CQD-assisted dsRNA enhanced gene-silencing efficiency and disease suppression against Phytophthora infestans and several fungal pathogens compared with naked dsRNA (Kostov et al., 2022). More recently, morphologically tuned graphitic carbon nitride (g-C_3_N_4_) platforms improved dsRNA loading/release and enhanced antiviral protection against tobacco mosaic virus without detectable phytotoxicity (Wei et al., 2025). Collectively, these studies positioned carbon-based materials as flexible carriers that can increase the effective intracellular dose and, in some systems, extend functional protection windows. For example, Delgado-Martín et al. (2022) reported approximately 50-fold higher internal dsRNA accumulation and approximately 13-fold higher derived siRNA levels in cucumber after spraying CD–dsRNA nanocomposites relative to naked dsRNA, directly supporting the translational relevance of these platforms (Delgado-Martín et al., 2022; Wang et al., 2025).

From a translational reporting standpoint, carbon-based delivery studies are the most informative when quantifying internalized dsRNA, siRNA output, and the duration of protection under realistic environmental stressors (e.g., UV exposure, temperature variability, hydration cycles, and rainfall), allowing improvements in delivery and movement to be distinguished from simple increases in surface persistence (Venu et al., 2025).

#### 3.3.4 Polymer-based complexes and adjuvant systems

Even without dedicated nanocarriers, formulation chemistry strongly determines whether the sprayed dsRNA reaches the bioactive sites. Spreader-stickers, surfactants, and humectants influence droplet spreading, evaporation, adhesion, and rehydration, thereby affecting the residence time on foliage and access to natural entry routes. Studies integrating LDH carriers with adjuvant packages illustrate that tuning these parameters can increase foliar uptake, systemic movement, and the duration of RNAi-based protection (Mitter et al., 2017; Jain et al., 2022).

When polymeric complexation is used (e.g., cationic polyplexes or interpolyelectrolyte complexes), the performance should be evaluated along the coupled efficacy and exposure dimensions. Cationic polymers can bind to dsRNA, condense it, and shield it from nucleases in plant and environmental matrices. Whitfield et al. (2018) compared linear and star-shaped cationic polymers and demonstrated strong dsRNA binding, protection against soil degradation, and controlled time-dependent self-release, illustrating how the polymer architecture and charge density govern both physical behavior and biological performance. In the foliar context, these benefits must be balanced against the need for timely uptake and bioavailability (Bachman et al., 2020; Wang et al., 2025).

Because stabilizing formulations can also extend persistence, they inevitably alter the environmental exposure windows. Bachman et al. (2020) emphasized that dsRNA typically dissipates rapidly across soil, water, and plant compartments, while noting that formulations can modify degradation kinetics and thus, exposure duration. Similarly, regulatory frameworks identify formulation as a core determinant of environmental fate and non-target risk (De Schutter et al., 2022). Consistent with this, a review by Luo et al. (2024) underscores that extended persistence, whether due to polymer encapsulation, inorganic matrices, or other stabilizers, can shift the compartments and organisms that experience meaningful exposure. Accordingly, formulation choices that improve efficacy should be co-designed with an explicit understanding of environmental fate and non-target hazards and reported in sufficient detail (polymer class, molecular weight, charge ratio, and spray parameters) alongside uptake, siRNA generation, rainfastness, and degradation kinetics to enable integrated interpretation (Whitfield et al., 2018; De Schutter et al., 2022; Luo et al., 2024).

Taken together, these studies indicate that naked dsRNA can be effective in receptive systems, but its protection window is often constrained by rapid loss of functional exposure after spraying. Formulated dsRNA improves performance when the main bottleneck is persistence, access to hydrated microenvironments, or intracellular delivery, although different platforms address these barriers to different extents. These observations support considering formulation not as an auxiliary feature, but as a central determinant of field-relevant SIGS performance.

## 4 Target selection and dsRNA design: determinants of SIGS performance

**Figure 2:**
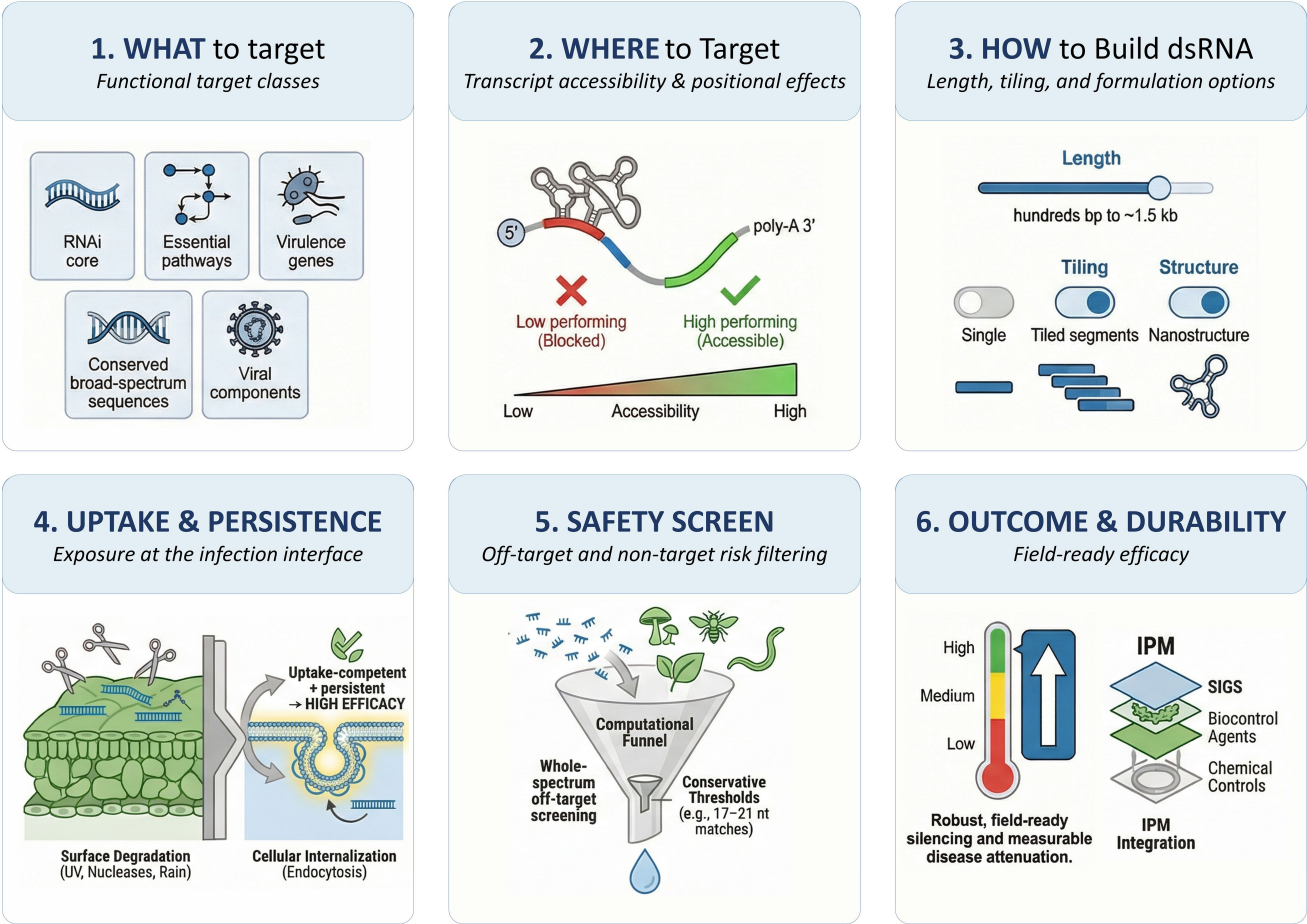
Mechanistic design framework for SIGS efficacy. Six coupled determinants shape SIGS performance from construct concept to field-ready outcomes: (1) What to target, prioritizing functional gene classes (RNAi-core, essential pathways, virulence determinants, conserved broad-spectrum sequences, and viral components); (2) Where to target, selecting transcript regions with higher local accessibility to support productive siRNA–mRNA pairing; (3) How to build dsRNA, tuning construct length, tiling/multisite coverage, and structure/formulation options that influence siRNA yield, stability, and bioavailability; (4) Uptake & persistence, the central gate in which environmental stability and exposure at the infection interface determine whether uptake-competent pathogens internalize RNA for intracellular processing; (5) Safety screen, whole-spectrum assessment of the expected siRNA pool to minimize off-target and non-target risks (typically prioritizing ≥17–21 nt perfect matches and evaluating near-matches case-by-case); and (6) Outcome & durability, integrating SIGS with durability-by-design (multi-targeting, sequence updating/rotation) and IPM to achieve robust disease attenuation under field conditions.

The SIGS field is increasingly moving beyond proof-of-concept demonstrations toward mechanism-informed design rules that may help predict when SIGS will work, why it will work, and how to build constructs that remain effective under realistic conditions. Across fungi, oomycetes, and viral pathosystems, performance is most consistently explained by three coupled variables: (i) which gene is targeted, (ii) where the dsRNA is directed within that transcript, and (iii) how the dsRNA is engineered (length, tiling, multisite coverage, and higher-order structure). These variables interact with the frequently underappreciated constraints, pathogen RNA uptake competence, and translational priorities, including off-target risk and durability (escape/resistance management) (Qiao et al., 2021; Koch et al., 2019; Song et al., 2018). These coupled determinants are summarized in a mechanism-informed design workflow (**Figure 2**). Importantly, these determinants are not independent: target-class choice influences the expected specificity burden, transcript-region choice affects both efficacy and off-target filtering, and persistence-enhancing formulations can modify the extent and duration of non-target exposure.

A key lesson from the case studies is that the strongest demonstrations do not simply show disease reduction; they implicitly reveal the design logic. For example, CYP51-derived dsRNAs (CYP3RNA) against *F. graminearum* established essential-pathway targeting as a reliable “anchor” strategy (Koch et al., 2016, 2019), whereas segment screens within single transcripts (e.g., *Myo5* in *Fusarium asiaticum*) showed that target position can dominate outcomes even when the gene is identical (Song et al., 2018). Similarly, antiviral SIGS studies converge on replicase/movement/coat protein targets and emphasize prophylactic timing, particularly when viruses encode strong suppressors of silencing (Kaldis et al., 2018; Worrall et al., 2019; Holeva et al., 2021).

### 4.1 Target classes and when they are most informative or effective

#### 4.1.1 RNAi-pathway targets as sensitizers

Targeting the pathogen’s own RNA silencing machinery (DCL/AGO and related nodes) has emerged as a sensitization strategy. Weakening the RNAi-core function can magnify phenotypes from otherwise moderate targets and strengthen multi-target designs. In fungi, dsRNAs targeting AGO/DCL homologs reduce disease in *F. graminearum*, supporting RNAi-core genes as practical vulnerabilities when uptake is competent (Werner et al., 2020). In oomycetes, targeting *Plasmopara viticola* DCL-like genes reduced sporulation and disease severity, consistent with cross-taxon relevance of this class (Haile et al., 2021). However, outcomes are contingent on environmental RNA uptake competence and potential redundancy among RNAi components across species (Werner et al., 2020; Qiao et al., 2021).

Targets of core metabolism and cell integrity often yield the most consistent growth and virulence penalties upon silencing. Sterol biosynthesis is a canonical example; dsRNAs derived from *F. graminearum* CYP51 genes suppress Fusarium head blight and reduce mycotoxin contamination, establishing an essential pathway targeting a robust anchor approach (Koch et al., 2016, 2019). Essential gene nomination frameworks tailored to SIGS reinforce this principle (Kim et al., 2023). Because some essential pathways have a long history of chemical selection (and known evolutionary plasticity), the design implication is not to avoid essential targets, but to deploy them with durability-by-design (e.g., multi-node targeting, rotations, or updates) rather than as single-target interventions. For example, the CYP3RNA strategy against *Fusarium graminearum* targeted multiple *FgCYP51* paralogs within the sterol biosynthesis pathway rather than relying on a single node, illustrating how broader pathway coverage can improve robustness and reduce dependence on a single target sequence (Koch et al., 2019; Rank and Koch, 2021).

#### 4.1.2 Virulence and infection-stage targets

Virulence determinants (effectors, infection-structure formation genes, and transcriptional regulators of pathogenicity) can provide high specificity and may reduce selection pressure relative to essential viability genes because fitness costs are concentrated during infection rather than during saprophytic growth. SIGS, used as a functional screen, highlights the value of this class when the application timing overlaps with the relevant expression window (Mosa and Youssef, 2021; Jin et al., 2025). The main limitation is stage specificity; if dsRNA is applied outside the key infection window or degrades quickly on the surface, even excellent virulence targets can underperform in more field-like settings. For example, topical antiviral SIGS is generally most effective when applied prophylactically or very early, whereas delayed application after infection establishment often gives substantially weaker protection because viral replication can outpace silencing (Holeva et al., 2021; Worrall et al., 2019; Hoang et al., 2022; Chen et al., 2025).

#### 4.1.3 Conserved broad-spectrum targets

Highly conserved cytoskeletal/trafficking genes can enable broad-spectrum suppression, but conservation increases the similarity to non-target eukaryotes, including beneficial fungi. β2-tubulin targeting demonstrated multi-pathogen protection and reduced fungicide resistance, illustrating feasibility when specificity is carefully managed (Gu et al., 2019). Accordingly, these targets are the most defensible only when paired with whole-spectrum off-target screening and, where possible, the selection of pathogen-biased regions that minimize similarity to host transcripts and beneficial eukaryotic orthologs, because their potential breadth of efficacy is accompanied by a higher specificity burden (Beernink et al., 2024; Zarrabian and Sherif, 2024).

#### 4.1.4 Antiviral SIGS targets

In antiviral SIGS, the sprayed dsRNA is primarily processed using a plant RNAi system to generate siRNAs that suppress viral RNAs. The effective target classes include replicase-associated regions, movement proteins, and coat proteins (Kaldis et al., 2018; Worrall et al., 2019; Holeva et al., 2021; Vadlamudi et al., 2020). Viral suppressors of RNA silencing (e.g., HC-Pro, 2b) are mechanistically attractive targets because suppressor knockdown can restore host antiviral RNAi, and multiple systems show strong prophylactic benefits when suppressors are targeted (Holeva et al., 2021; Kaldis et al., 2018; Vadlamudi et al., 2020). In practice, antiviral SIGS performs best when used prophylactically or very early, as established infections may outpace silencing, depending on viral kinetics and suppressor strength. For example, topical dsRNA targeting the 2b and CP genes of *Cucumber mosaic virus* protected plants against local and systemic infection when applied preventively, illustrating the advantage of early deployment before viral accumulation becomes established (Holeva et al., 2021; Choi et al., 2026)practice.

#### 4.1.5 Host susceptibility genes

An emerging extension is the transient silencing of plant susceptibility genes as a non-transgenic analog to S-gene editing, potentially enabling broad-spectrum outcomes when pathogen diversity is high or when pathogens rapidly escape sequence-specific targeting. Because host-directed designs can carry direct phenotypic and agronomic trade-offs in the crop itself, they require the strictest safety threshold among SIGS target classes, with explicit risk–benefit framing and careful evaluation of unintended host effects (Touzdjian Pinheiro Kohlrausch Távora et al., 2022; Syed et al., 2025).

### 4.2 Transcript-region choice and construct architecture

The central maturation point for SIGS is when gene identity ceases to be predictive. Different regions within the same transcript can yield sharply different outcomes because silencing requires productive siRNA–mRNA pairing and local accessibility for RISC engagement. In *F. asiaticum*, dsRNAs spanning different *Myo5* segments produce strong segment-dependent phenotypes and suppress virulence, providing a clear experimental basis for positional effects as a dominant design variable (Song et al., 2018).

Accordingly, recent protocol-oriented literature increasingly treats region screening and tiling as best practices rather than late-stage optimization (Song et al., 2018; Koch et al., 2019). Mechanistically, positional performance is often influenced by accessibility, avoiding regions prone to strong local structures or otherwise reduced RISC access, which can hinder effective siRNA binding. More broadly, RNAi design discussions emphasize that the sequence context and local structure can modulate apparent potency even when the targeted gene is unchanged (Chen et al., 2025). Treating transcript region choice as a primary design axis is especially important in new pathosystems where uptake competence and dominant processing routes remain uncertain (Qiao et al., 2021; McLoughlin et al., 2018). Current syntheses converge on dsRNA length as a trade-off rather than a “longer is always better” relationship. Many effective foliar SIGS constructs fall in the hundreds of base pairs up to ~1.5 kb, balancing siRNA yield with uptake, manufacturability, and handling constraints; very long dsRNAs can generate more siRNAs, yet still underperform if uptake is limited (Koch et al., 2019; Song et al., 2018; Delgado-Martín et al., 2022). Flagship demonstrations, such as CYP3RNA, have benefited from dense siRNA production and broad intra-transcript coverage (Koch et al., 2019), but current guidance increasingly recommends testing multiple construct lengths for the same region rather than committing to a single length *a priori* (Mosquera-López et al., 2025; Pratama et al., 2026).

Tiling and multisite coverage improve robustness by distributing siRNA across multiple high-performing regions, reducing the risk that a single poorly accessible segment becomes a bottleneck and increasing resilience to pathogen sequence variation (Chen et al., 2025; Mosquera-López et al., 2025). This coverage logic also aligns with antiviral systems, where abundant siRNA production can support mobile silencing signals and extend protection profiles (Kaldis et al., 2018; Vadlamudi et al., 2020).

Beyond linear design, higher-order RNA structure is emerging as a lever: carrier-free RNA nanostructures can enhance stability and efficacy, expanding the design space beyond classic “sequence + length” paradigms (Wu et al., 2024; Zhao et al., 2024). Importantly, structure-based gains intersect with formulation-enabled delivery (Section 3): by shifting persistence and bioavailability, formulations can change which architectures appear “optimal” for the same target (Chen et al., 2023; Xi et al., 2025).

### 4.3 Uptake, persistence, specificity, and durability as co-determinants of performance

Cross-species comparisons show that SIGS outcomes are frequently limited by the pathogen’s ability to efficiently internalize exogenous RNA. For example, Qiao et al. (2021) showed that pathogens such as *Botrytis cinerea, Sclerotinia sclerotiorum, Rhizoctonia solani, Aspergillus niger*, and *Verticillium dahliae* displayed efficient dsRNA uptake, whereas uptake was weak or absent in *Colletotrichum gloeosporioides* and limited in *Phytophthora infestans*, with corresponding differences in SIGS efficacy. Mechanistic evidence for endocytosis-mediated uptake in *Verticillium dahliae* further supports the view that uptake is an active regulatory process rather than passive diffusion (Qiao et al., 2021; Šečić and Kogel, 2021; He et al., 2024; Liu et al., 2025).

Uptake limitations are also environmental; nuclease activity and surface-associated degradation can reduce the effective dose before internalization, making persistence and protection key co-determinants of efficacy (Hoang et al., 2022; Mamun et al., 2025; Pratama et al., 2026). This yields an integration rule that aligns directly with Section 3: Target selection and dsRNA architecture should be co-designed with the delivery strategy. In uptake-competent fungi (e.g., systems in which dsRNA is detected in hyphae), region choice and architecture often dominate the outcomes (McLoughlin et al., 2018; Nerva et al., 2020). In uptake-limited contexts, formulation has become the primary bottleneck, and multiple delivery strategies, including clay-based matrices, nanocarrier systems, and artificial nanovesicles, have improved the efficacy (Niño-Sánchez et al., 2022; Qiao et al., 2023; Spada et al., 2025; Worrall et al., 2019). CaP nanoparticles that enhance the effects of dsRNA on growth and mycotoxin production in *F. graminearum* provide another example of how engineered delivery can shift the biological outcomes for a given target set (Stakheev et al., 2025). Collectively, these findings reinforce the practical conclusion that “good targets” can fail without alignment between uptake and persistence, whereas “moderate targets” can become field-relevant when delivery constraints are removed (Chen et al., 2023; Mamun et al., 2025; Xi et al., 2025).

### 4.4 Specificity and non-target risk assessment

As SIGS approaches broader deployment, biosafety scrutiny increasingly focuses on unintended silencing in host plants and non-target organisms exposed on plant surfaces, including beneficial fungi and microbiome-associated eukaryotes. This scrutiny intensifies when formulations increase persistence, because efficacy gains can coincide with longer non-target exposure windows (Beernink et al., 2024; Zarrabian and Sherif, 2024; Mamun et al., 2025).

Current literature increasingly supports the use of whole-spectrum off-target screening, in which the full set of potential siRNAs generated from dsRNAs is computationally evaluated rather than relying on only a small subset of top-ranked candidates. Tools such as dsOMG operationalize this approach and support more defensible specificity arguments for both reviewers and regulators (Lyu et al., 2025). In parallel, machine learning approaches (e.g., AttSiOff) highlight a growing direction for jointly predicting the inhibition potential and off-target risk (Liu et al., 2024a). Practical criteria emphasized across RNAi safety discussions include avoiding long uninterrupted matches to non-target transcripts and applying conservative thresholds when designing broad-spectrum targets (Lü et al., 2022; Lyu et al., 2025).

Chemical modification is sometimes discussed as an additional risk-control lever; for example, backbone or terminal modifications can be used to alter RNA stability or reduce unintended interactions. However, such choices must be balanced against the cost and potential impacts on processing and efficacy in relevant biological systems (Hu et al., 2026). For translational clarity and reproducibility, it is increasingly important to report the dsRNA coordinates, in silico filtering rules, and the expected siRNA spectrum, particularly as regulatory guidance continues to evolve (Gathmann et al., 2025). In practical terms, target selection, construct design, and biosafety assessment should therefore be treated as a single iterative workflow: candidate regions should be chosen not only for expected silencing potency, but also for acceptable off-target profiles across the full predicted siRNA spectrum.

### 4.5 Designing for durability: escape and resistance pathways

RNAi-based interventions can impose selection pressures via multiple routes, including sequence variations at target sites, altered uptake, enhanced degradation, and shifts in RNAi processing; therefore, durability planning should begin at the target-selection stage rather than after efficacy loss is observed (Rank and Koch, 2021; Beernink et al., 2024). Case studies for application have already predicted the durability logic: antiviral systems repeatedly show that timing and persistence determine whether viral replication outruns silencing (Worrall et al., 2019; Holeva et al., 2021), and field demonstrations against viruses highlight the need for strategies that remain effective under heterogeneous epidemic pressure (Patil et al., 2021).

Recent experimental demonstrations support multi-targeting as a practical strategy for increasing the evolutionary barrier to escape. For example, dual- or multi-gene targeting has been reported to strengthen silencing outcomes compared with single-target designs in experimental systems, supporting its use as a durability-oriented strategy rather than simply a way to increase dsRNA load (Pant and Kaur, 2023; Jin et al., 2025; Yan et al., 2025). This same logic motivates rotation or sequence updating, particularly for pathogens with high genetic variation or rapid population turnover (Rank and Koch, 2021; Bocos-Asenjo et al., 2025).

Delivery innovation is durable because persistence-enhancing formulations can stabilize performance under environmental stress and reduce the need for extreme dosing, potentially improving consistency without proportionally increasing exposure (Niño-Sánchez et al., 2022; Mamun et al., 2025; Xi et al., 2025). Finally, placing SIGS within integrated pest management remains the most coherent durability framework, aligned with broader risk/benefit considerations and evolving regulatory landscapes (Touzdjian Pinheiro Kohlrausch Távora et al., 2022; Gathmann et al., 2025).

Across systems, the performance is most predictable when the target choice, transcript region, and construct architecture are selected under explicit assumptions regarding uptake competence and environmental persistence. In uptake-competent pathogens, region/tiling choices often dominate; in uptake-limited contexts, delivery becomes the main determinant of realized silencing. These same choices set the boundary conditions for non-target risk and durability, making specificity screening and multisite designs part of the construct design rather than *post-hoc* validation.

## 5 Conclusions and future perspectives

Spray-induced gene silencing is no longer defined primarily by whether exogenous dsRNA can reduce disease in controlled assays but by the emerging ability to predict and engineer performance under realistic conditions. The evidence synthesized here supports a simple organizing principle: SIGS is an exposure-limited intervention whose success depends on the alignment of mechanistic biology with construct engineering. In practice, efficacy is governed by a small set of coupled determinants—functional target class, within-transcript target position, and dsRNA architecture—that operate under the overriding constraint of pathogen RNA uptake competence. These variables explain why “good” targets can fail when RNA is not delivered to, retained within, and encountered at the relevant infection interfaces, and why moderately performing targets can become field-relevant once exposure barriers are reduced.

This framing has immediate implications for translation. Formulation and delivery should be treated as design variables, equally important as target selection, as they set the temporal and spatial windows during which dsRNA and derived siRNAs remain bioavailable. Persistence-enhancing carriers can extend protection and stabilize outcomes under weathering and nuclease pressure; however, they can also shift the apparent optimality of target regions and architectures by changing where and for how long RNA is accessible. Consequently, target rules and delivery choices should be developed as a single design problem rather than as sequential steps, with the construct design evaluated under the same exposure constraints anticipated for deployment.

The maturation of SIGS also underscores that target selection is not a single decision but a workflow. In the revised perspective presented here, future progress will depend on moving from proof-of-concept targeting toward more standardized and comparative design pipelines. Robust strategies should begin with function-informed nomination of targets (e.g., essential pathways, infection-stage determinants, or RNAi-core sensitizers), followed by within-transcript region selection and empirical tiling to identify high-performing segments. Construct architecture—length, multisite coverage, and structure—becomes a durability and consistency tool, enabling redundancy across transcript regions and buffering against sequence variation or localized accessibility constraints. In parallel, standardized uptake-competence and exposure readouts should be incorporated more routinely alongside disease endpoints to explain success and failure across pathosystems and to improve cross-study comparability.

Priority needs for the field now include standardized assays for pathogen RNA-uptake competence, formulation benchmarking under realistic weathering conditions, fuller reporting of dsRNA coordinates and predicted siRNA spectra, and durability-oriented designs that integrate multisite targeting with broader disease-management frameworks.

SIGS deployment is also judged based on its specificity and durability. Construct-level specificity arguments are increasingly expected to account for the full spectrum of siRNAs generated from dsRNAs, and transparent reporting of dsRNA coordinates and *in silico* filtering criteria are becoming essential for reproducibility and regulatory assessments. Similarly, durability should be incorporated at the design stage through multisite coverage, multi-targeting, and, where needed, sequence updating or rotation, acknowledging that loss of efficacy can arise through target variation, changes in uptake, enhanced degradation, or altered RNAi processing. Taken together, the field is moving toward a mechanistic and engineering discipline in which reliable SIGS performance can be achieved by co-optimizing exposure control, target and construct design, uptake constraints, and durability-by-design within integrated disease management programs.
